# Inferring Regional-Scale Species Diversity from Small-Plot Censuses

**DOI:** 10.1371/journal.pone.0117527

**Published:** 2015-02-23

**Authors:** John Harte, Justin Kitzes

**Affiliations:** Energy and Resources Group, University of California, Berkeley, California, United States of America; University of Sydney, AUSTRALIA

## Abstract

Estimation of the number of species at spatial scales too large to census directly is a longstanding ecological challenge. A recent comprehensive census of tropical arthropods and trees in Panama provides a unique opportunity to apply an inference procedure for up-scaling species richness and thereby make progress toward that goal. Confidence in the underlying theory is first established by showing that the method accurately predicts the species abundance distribution for trees and arthropods, and in particular accurately captures the rare tail of the observed distributions. The rare tail is emphasized because the shape of the species-area relationship is especially influenced by the numbers of rare species. The inference procedure is then applied to estimate the total number of arthropod and tree species at spatial scales ranging from a 6000 ha forest reserve to all of Panama, with input data only from censuses in 0.04 ha plots. The analysis suggests that at the scale of the reserve there are roughly twice as many arthropod species as previously estimated. For the entirety of Panama, inferred tree species richness agrees with an accepted empirical estimate, while inferred arthropod species richness is significantly below a previous published estimate that has been criticized as too high. An extension of the procedure to estimate species richness at continental scale is proposed.

## Introduction

The capacity to predict patterns in the abundance and distribution of species across multiple spatial scales and across a wide diversity of taxonomic categories and habitats is of value both in biological conservation and in advancing our fundamental understanding of biological diversity. Among the many issues that such capacity would illuminate are the extent to which rare species are present in an ecosystem and the numbers of species at spatial scales larger than those that can be censused directly. A recent comprehensive census of tropical arthropods and trees [1,2] in small plots in the San Lorenzo Protected Area (SLPA), a forest reserve in Panama, provides a unique opportunity to both test and apply macroecological theory. Here we test and apply an inference procedure [3] based on the maximum entropy (MaxEnt) concept for up-scaling both arthropod and tree species richness from small plots to regional scale.

**Fig 1:**
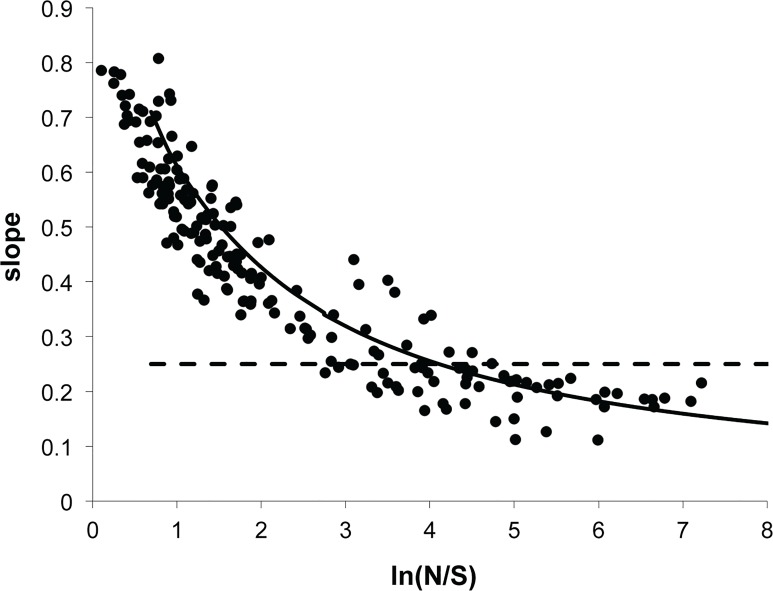
The species-area relationship in the Maximum Entropy Theory of Ecology. Predicted and observed values of the local slope of the SAR are plotted as a function of the local value of the ratio of abundance to species richness. Data are derived from SARs for a variety of taxa, habitats, and spatial scales from multiple sources; a derivation of the theoretical prediction from MaxEnt and sources of data are provided elsewhere [4]. Note that the often-assumed power-law form of the SAR with slope ∼0.25 would correspond to all data points lying on a horizontal line at the 0.25 mark on the vertical axis.

Critical to our method of up-scaling species richness is accurate prediction of the form of the species-area relationship (SAR). Fig. 1 shows the predicted form of the SAR: At any spatial scale the slope of log species richness versus log area is predicted to be a unique function of the ratio of the number of individuals to the number of species at that scale [4]. Based on this result, extrapolation of species richness from small plots to much larger areas is an iterative procedure. Python code to carry out the calculations is provided as Supporting Information (S1 Script) and the explicit equations are given in [3,4].

Confidence in the underlying theory is established by four independent tests. First, we refer to Fig. 1, showing that SAR data across a wide range of spatial scales, taxonomic groups, and habitats follow reasonably well the predicted scale-collapse form for that metric. Second, in previous work [4] we showed that the up-scaling procedure accurately predicts tree species richness at the scale of the Western Ghats in India (60,000 km^2^) based on census data from 48 plots, each ¼ ha, scattered throughout the Ghats. Our MaxEnt-based procedure predicts an answer within 10% of the known value, which is approximately half the expected standard deviation in predicted up-scaled species richness that results from the empirical spread in slope values shown in Fig. 1. Third, we show here that the theory predicts the species abundance distribution (SAD) for trees and arthropods within the SLPA census plots, and in particular very accurately captures the rare tail of the observed distributions. The rare tail is emphasized both because of the conservation value of knowing the numbers of rare species and because the shape of the species-area relationship is especially influenced by the numbers of rare species. Finally, we show here that the up-scaling procedure predicts an estimate of tree species richness at the scale of Panama that agrees with previous and very plausible direct empirical estimates.

Most existing theories that attempt to predict patterns in macroecology range along a continuum that extends from purely phenomenological approaches in which observed patterns are parameterized and extrapolated (for example [5] and [6]), to approaches in which selected subsets of ecological mechanisms are embodied in parameterized models (for example [7]). The framework adopted here [4] is not located on that continuum. Based upon empirical state variables describing the total number of species and individuals at some spatial scale, it makes no explicit assumptions about dominant mechanisms and contains no adjustable parameters. It is based on a rigorously proven inference method derived from information theory [3,8].

The essential idea is that the MaxEnt inference procedure yields the least-biased prediction of the shape of a probability distribution that is constrained by prior knowledge [9]. In the application to ecology, prior knowledge consists of measured values of state variables (for example, the total number of species and individuals at some spatial scale constrains the species-abundance distribution, much like pressure, volume and temperature of a gas constrain the distribution of molecular velocities in statistical physics). From knowledge of the state variables, the procedure predicts the shapes of most of the metrics of macroecology including, at the community level, the rate of increase of species richness with area sampled (the species-area relationship or SAR), the distribution of abundances across species (the species-abundance distribution or SAD), and at the species level, a measure of the aggregation of individuals across space (the species-level spatial abundance distribution or SSAD). Numerous tests of these and other predictions of the MaxEnt-based approach have been carried out for a wide variety of taxonomic categories and habitats [3,4,10,11]. We describe the method more fully in S1 Text, and additional detail is available elsewhere [3].

Despite past success of this MaxEnt method for up-scaling tree species richness, the availability of census data that would permit application to tropical arthropods has heretofore not been available. The SLPA census data provide the opportunity to apply the inference procedure to estimate the total number of arthropod and tree species at spatial scales that include the 6000 ha SLPA as well as all of Panama, with input data only from censuses in twelve 0.04 ha plots.

Below we estimate arthropod species richness at the scale of the reserve and of Panama, and compare to previous estimates [1,12]. We also examine the sensitivity of inferred species richness at large spatial scales to choices of taxonomic categories and determine the degree to which our results depend on whether up-scaling is carried out separately order by order, or guild by guild, or for all orders combined. Finally, in a more speculative vein, we suggest a way to extend species richness predictions to even larger, continental, spatial scales, and infer arthropod species richness in all of Amazonia.

## Materials and Methods

Recently Basset et al. [1,2] published results from an intensive and spatially explicit tree and arthropod census in tropical forest habitat within the San Lorenzo Protected Area (SLPA) a forest reserve in Panama. Because a wide range of arthropod collection methods are used [2], and because sampling is carried out from the soil to the forest canopy, the data set uniquely provides detailed abundance information across the taxonomic breadth of the Arthropoda. To our knowledge, it is the most thorough and reliable data set available that can be used, within our theoretical framework, for estimating arthropod species richness at spatial scales larger than those directly censused.

The data are collected from 12 plots, each of area 0.04 ha and distributed over the 6000 ha forest reserve. A total of 6144 arthropod species divided among 129,494 individuals were detected in the 12 plots. A total of 163 tree species divided among 1,556 individuals with greater than 10 mm dbh were also detected. Plot-level averages of species richness and abundance for trees and for each arthropod order are given in Table 1, as well as values for ten guilds that are delineated in four arthropod orders by Basset et al. [1] based on either trophic behavior or taxonomy.

**Table 1:** Second and third columns give values of species richness and abundance for each taxonomic category: each of 13 orders, all orders combined, ten guilds, and trees at the scale of 0.04 ha.

Category	*S*	*N*	Percent of plots best fit by log series SAD
ORDER			
Acari	87	2015	100
Araneae	76	184	100
Blattodea	17	59	100
Coleoptera	725	50144	100
Collembola	31	1755	50
Diptera	41	232	100
Hemiptera	116	588	100
Hymenoptera	224	1349	100
Isoptera	23	433	90.9
Lepidoptera	143	322	100
Opilliones	12	36	81.8
Orthoptera	29	142	83.3
Psocoptera	9	18	66.7
LUMPED ORDERS	1536	12155	100
GUILDS			
Coleoptera predators	236	1135	100
Coleoptera scavengers	44	220	100
Coleoptera fungal feeders	168	1266	100
Coleoptera chewers	259	215050	100
Diptera predators	36	15	100
Diptera scavengers	5	13030	83.3
Orthoptera chewers	9	215	75
Orthoptera scavengers	22	1616	83.3
Hymenoptera ants	147	1127	100
Hymenoptera bees	188	17070	87.5
TREES	47	133	91.7

Values represent plot averages and are rounded to nearest integer. The Fourth column gives the percentage of plots that are best described by the log series SAD compared to the lognormal.

These data are used here to test the predicted species-abundance distribution (SAD), separately for trees and for arthropods, both using the plot-scale data and the combined data from all plots. In the case of the arthropods, we conducted these tests for the species in each order, for the species in each delineated guild, and for the species in all orders combined. These tests provide insight into the spatial and taxonomic domain of the theory. The predicted log series SAD is uniquely determined from the two measured state variables in each plot: number of species, *S*, and total number of individuals, *N*. If mean abundance is constrained by knowledge of *S* and *N*, fitting the lognormal introduces one additional parameter for each plot: the variance of the distribution. We use AIC to compare the validity of the predicted SAD with that of the lognormal distribution.

To more strongly justify the validity of the theory underlying the up-scaling procedure, we examine how well the log series abundance distribution captures the rare tail of that distribution, which is particularly important in shaping the SAR and therefore strongly influences the outcome of the up-scaling. Hence we tested just the predicted rare tail of the distribution for the same cases as above. In our theory, the ratio of N/S determines the shape of the species abundance distribution, which in turn determines the number of rare species at any scale and thus strongly influences the slope of the SAR at that scale. Equation S-8 in S1 Text results in an SAR that is strongly influenced only by the rare tail of the SAD.

Our approach to up-scaling utilizes the scaling law illustrated in Fig. 1 and discussed further in S1 Text. This method requires that the small-scale data set that is the starting point for up-scaling be from a contiguous area, so the analysis is done with values of the state variables *S* and *N* at the 0.04 ha scale. Our species-area based approach [3,4] is theory-driven, with the shape of the SAR determined only from these state variables and the MaxEnt theory of ecology. This approach is thus extremely restrictive, allowing only one possible deterministic predicted value of large-scale richness for any given *S* and *N* combination. This approach contrasts with common and more flexible methods based on statistical estimators, where the choice of a parametric or non-parametric method and its parameterization can greatly influence predictions of large scale richness [13]. Despite its very restrictive nature, we have previously demonstrated the success of this theory-driven approach [4], and we test it further with tropical tree data below before applying it to arthropod data.

We estimate tree and arthropod species richness in the 6000 ha reserve by up-scaling the 0.04 ha observational data. For the arthropods, we do this at three taxonomic levels of resolution: each order separately, separate trophic guilds within orders, and all arthropods lumped together. For the trees, we perform a single up-scaling calculation.

For the separate order analysis, we calculate the mean *S* and *N* for each order across the available plots, upscale each order separately, and sum the predicted richness across orders at each larger scale. In calculating the mean *S* and *N* for each order, we note that the methods used by Basset et al. [1,2] to census arthropods varied across the twelve plots. As we do not wish to make any assumptions about the relationship between taxonomic group, sampling method, sampling intensity, and observed richness, we do not apply any general or formal correction to Bassett’s reported data to account for this variation. However, in one particularly significant effect, we note that in several plots, most notably the F1, F2, and F3 plots that were intended as fogging surrogates for the C1, C2, and C3 plots [2], zero individuals were recorded within several otherwise abundant orders. To correct for this likely undercounting, we ignore the zero counts of the orders Acari, Collembola, and Lepidoptera in the plots F1, F2, F3, and R2 when calculating the mean *S* and *N* for these orders, taking these to be false absences due to sampling method. While this adjustment affects our order-level and plot-level (see below) predictions, it leads to a discrepancy of less than 5% in our main lumped arthropod richness predictions (S1 Table).

For the separate guild analysis, we similarly calculate the mean *S* and *N* for each guild, including a relatively large “Other” guild (approximately 40% of all individuals), across the available plots, upscale each guild separately, and sum the predicted richness across guilds at each larger scale. We make no adjustments to the raw data reported by Bassett et al. for this analysis. For the lumped arthropod predictions, we sum the mean *S* and *N* for all orders at the 0.04 ha scale, given the above data adjustments, and up-scale these summed variables directly. For the tree predictions, the mean *S* and *N* across plots is up-scaled directly.

To gain information on the uncertainty in the up-scaled predictions, we additionally up-scale tree and lumped arthropod richness using the individual plot values of *S* and *N*, where zero values for order-plot combinations described above were replaced with the mean values for that order across remaining plots to avoid potentially significant underestimation. We then calculate the mean and standard error of the predicted richness for trees and lumped arthropods at each scale.

## Results

Results from an AIC evaluation of the goodness of fit of the lognormal and the log series SAD are summarized in Table 1. Using data from the 13 arthropod orders, the analysis shows that plot by plot and order by order, out of a total of 130 plot-order combinations with state variables large enough to meaningfully test distributions (more than three species and ten individuals), 109 combinations are better described by the predicted log series. In no order were more than half of the plots better described by the lognormal SAD. In general, the log series prediction performs best for the more abundant orders. When all orders are combined, all plots are also better described by the log series. For the tree data, the log series SAD outperforms the lognormal in 11 of the 12 plots.

**Fig 2:**
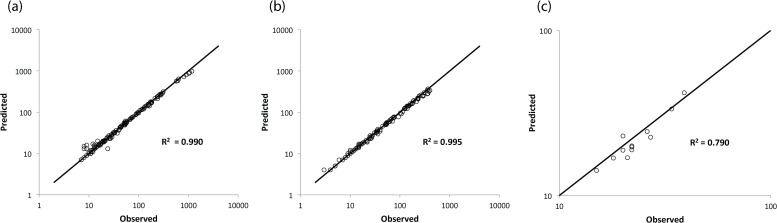
Predicted versus observed values of the number of arthropod species with fewer than ten individuals and singleton trees. Straight lines are one-to-one lines, and R^2^ values are calculated around this line. a. Arthropod order-plot combinations (n = 137). b. Arthropod guild-plot combinations (n = 106). c. Singleton trees in each plot.

A more detailed test of the validity of the predicted log series SAD is shown in Fig. 2 A-C. For arthropods, we compare observed and predicted numbers of species with fewer than 10 individuals; counting species with n < 10 is a reasonable but admittedly arbitrary choice for examining the capacity of theory to predict rarity. Fig. 2A tests the arthropod rarity prediction for each combination of order and plot, while Fig. 2B tests the arthropod rarity prediction for each combination of guild and plot. Fig. 2C tests the tree rarity prediction for each plot.

In all the cases considered in the figures, rarity is accurately predicted, with log-log R^2^ values, measuring the fraction of variation in observed values that is explained by the predicted values (S1 Text), quite close to 1. The comparable success of the predictions when orders are subdivided into guilds implies insensitivity in the abundance distribution to taxonomic categories used for analysis. We found further evidence for such insensitivity to the taxonomic level of analysis by examining predicted versus observed rarity with all arthropod orders lumped together rather than with orders subdivided into guilds. Moreover, an analysis similar to those in Fig. 2A-B except with all orders combined, indicates that for this arthropod data set the accuracy of the predicted SAD is quite insensitive to the choice of taxon categories.

**Fig 3:**
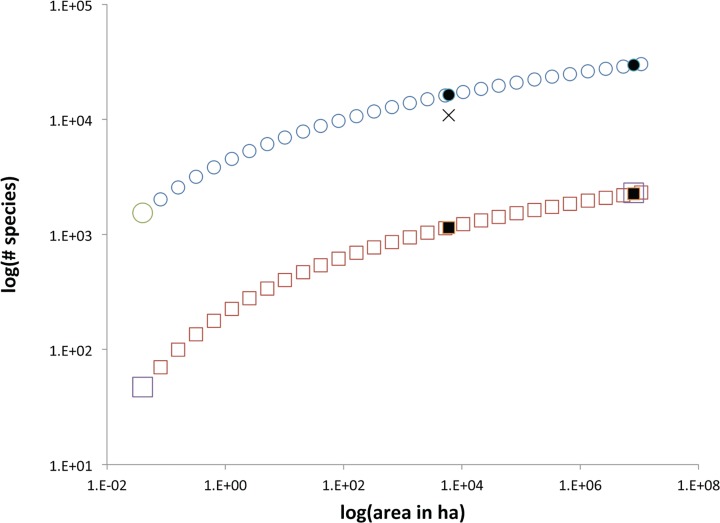
Predicted, measured, and estimated species richness for trees and arthropods. Small open circles are predicted arthropod diversity, small open squares are predicted tree diversity. Large open circle is measured arthropod diversity and large open squares are measured or estimated tree diversity. Filled circles are predicted arthropod diversity at the scale of the Reserve and of Panama, filled squares are predicted tree diversity at those same scales. The X marks the previous estimate for arthropod diversity at the reserve scale of Basset et al. [1]. Standard errors on estimates (S1 Table) are smaller than displayed marker size.

Fig. 3 shows the summarized up-scaling results for trees and arthropods, while S1 Table gives the numerical results of the species richness up-scaling for all arthropods lumped, each arthropod order, each arthropod guild, and all trees. All calculations are carried out at scales that are powers of two times the scale of observation; interpolation yields values at other scales. The lumped and separate guild predictions agreed to within 7% at the scale of Panama, while the lumped approach predicted 31% fewer species at this scale than the separate order predictions (S1 Table). For consistency with the approach of Bassett et al. [1], in both the figure and below, we report the summed richness of order-by-order up-scaling using mean values of *S* and *N* at the plot scale (Table 1). For trees, all species are combined into a single group for up-scaling purposes. Results based on dividing the data in different groupings are available in S1 Table.

Additionally, we calculate a coarse uncertainty measure for our estimates by constructing standard errors for species richness estimates for lumped arthropods and trees at each scale based on separate predictions from each of the 12 plots. For arthropods, these means of 12 up-scaled predictions differed from the lumped arthropod predictions beginning with a single mean *S* and *N* at small scales by less than 1%, while mean tree predictions differed by 11% at the scale of Panama (S1 Table).

Scaling up from the observations in the 0.04 ha plots, at the 0.48 ha scale (the combined area of the 12 0.04 ha plots) we predict a total of 4000 (SE 356) species of arthropod, which is approximately two-thirds of the combined species richness, 6144, in all 12 plots. This is expected because the 12 plots are not contiguous; observed distance-decay of species similarity across plots [1] implies that the total species richness of separated plots with a given summed area should contain more species than would a contiguous plot of that same area.

At 6000 ha, the area of the reserve, species richness is predicted to be approximately 22,700 (SE 1590) species. If the analysis is carried out by up-scaling from state variables characterizing guilds, the result at 6000 ha is approximately 16,800 species, while up-scaling based on lumping all orders at the smallest scale produces a result of 16,300. At this scale, we thus predict approximately twice the ∼11,000 focal arthropod species, the value comparable to our result, than had been inferred in Basset et al [1]. Moreover, whereas Basset et al. [1] concluded that 1 ha of the rainforest would contain more than 70% of the total richness in the 6000 ha reserve, we conclude (S1 Table, Fig. 3) that only 22% of the species in the 6000 ha reserve are present in a typical single hectare. Extending the analysis to larger spatial scales, we infer that the focal arthropod species in the 0.04 ha plots scale up to a total of 43,000 (SE 2870) species in an area equal to that of Panama (80,000 km^2^), and that of these, approximately one-third are in the order Coleoptera. Our estimate of arthropod species richness in Panama is less than Erwin’s estimate [12] for arthropod species richness in just one hectare of that nation’s tropical forest, and thus adds to the doubt that has been cast [14] on his estimation methods.

We infer approximately 1150 (SE 210) tree species at the 6000 ha scale of the reserve and 2260 (SE 440) species at the scale of Panama. This estimate is remarkably close to the estimate by Condit et al. [15] of 2300 tree species in Panama based on extensive surveys. From the estimate of tree species diversity at 50 ha scale, we conclude that the reserve contains, at that scale, almost twice as many species as does the well-studied Barro Colorado Island tropical forest plot [7]. Erwin [12] reports up to 245 tree species per ha in rich tropical forest, which is slightly higher than our interpolated value from S1 Table of 207 species at 1 ha scale.

We note that if species and individuals from spatially separated plots are aggregated and the analysis is carried out for all plots combined, at any of the taxonomic categories, the predictions are poorer than if the analysis carried out at plot scale. This is expected [3] because of observed [1] distance decay in species similarity across space.

## Discussion

From the dependence of the predicted SAR local slope *z* on the ratio of *N* to *S* in Fig. 1, it is clear that as area increases, the predicted slope decreases. The reason is that average total abundance, *N*, increases linearly with area whereas *S* increases sublinearly and so as area increases, one slides to the right along the x-axis in Fig. 1, and the slope of the SAR decreases. The increase of species richness with area is faster than logarithmic but slower than the conventionally assumed power law behavior. We note that if a power-law SAR with a slope of 0.25 is assumed, and the 0.04 ha census data are scaled up, 182,300 species of arthropods and 5600 species of trees are predicted in an area the size of Panama, both of which greatly exceed plausible estimates.

In a more speculative vein, if we naively extend the MaxEnt predictions for all species of arthropods and trees to even larger spatial scales than the area of Panama, we find that at sufficiently large scales the theory will significantly underestimate species richness. For example, at the scale of Amazonia, ∼ 5.5*10^6^ km^2^, we predict only about 20,000 species of Coleoptera and 56,00 species of arthropods, which is clearly an underestimate. Similarly, there are an estimated 16,000 species of trees in all of Amazonia [16], whereas extrapolation using Fig. 1 predicts only about 3020 species. A method for improving estimation of species richness at continental scales is suggested here.

The breakdown of our method at the scale of large biomes or continents is likely a consequence of habitat transitions that occur within such large areas, resulting in groups of sizeable numbers of species with non-overlapping ranges [17]. Related to this, our predicted decrease of slope with increasing area (Fig. 1) appears to conflict with observations showing that slope can increase at very large areas as habitat boundaries are crossed [5,18]. In our applications, MaxEnt yields least biased estimates of large scale species richness assuming prior knowledge of the values of the small-scale state variables. Additional prior knowledge beyond that contained in knowledge of the small-scale values of *S* and *N* would, however, result in a different up-scaling prediction and thus, within the MaxEnt framework, additional information can be used to improve estimation at very large scales.

Suppose, for example, that we know that within some large biome or continent, there were two distinct habitats with nearly entirely non-overlapping species. Then the MaxEnt formalism for up-scaling would yield a more accurate answer if species richness in each of the regions were separately up-scaled from the small-scale state variables and then the two results were added together to give the total species richness [17]. Applying this concept to Amazonia would entail censusing efforts such as those carried out by Basset et al. [1] in appropriate locations to sample the dominant distinct habitats and associated clusters of species. Knowing what constitutes a distinct habitat is of course a source of uncertainty, but this approach could yield a significantly more reliable estimate of large-scale diversity than is currently available.

To explore the possibility of implementing this strategy, consider the up-scaling of tree species richness to an area the size of Amazonia. Because the true answer is ∼ 16,000 species [16] we can work backwards to estimate the effective number of distinct habitats. Our inference procedure predicts only about 3020 species if we upscale directly to the area of Amazonia, so let us assume that there are *m* distinct subregions, which for simplicity we take to be of equal area in this example, in Amazonia. Because they are non-overlapping, each has to be upscaled separately from small scale data within those regions and then the results added together to get the total.

Solving *m* *S_trees_(*A*/*m*) = 16,000 to illustrate this concept, where S_trees_(*A*/*m*) is projected tree species richness in a fraction 1/*m* of the area, *A*, of Amazonia, we obtain *m* ∼ 6 and S(A/6) ∼ 2,700 as the unique solution. Hence we infer that there are roughly six distinct sub-regions that would need to be up-scaled separately in Amazonia to estimate tree diversity reasonably accurately. We emphasize that in this simple example we have assumed the subregions are of equal area but that assumption can be easily improved upon with biogeographic data.

That the number of sub-regions derived from this procedure turns out to be six is supported by previous biogeographical analyses. Within Amazonia, six bioregions are traditionally defined (Central Amazonia, Eastern Amazonia, the Guyana Shield, Southern Amazonia, Northwestern Amazonia and Southwestern Amazon); see, for example, figure 1 in [16]. This suggests that the MaxEnt upscaling method is in fact compatible with the observed tendency for SAR slopes to increase at very large area, provided the constraints arise from state variables that are suitably derived from empirical data on low- or non-overlapping sub-regions.

If those same sub-regions are sufficient to obtain a reasonably reliable estimate of Amazonian arthropod diversity, then we infer a total of 6 * *S*_arthropod_(5.5*10^6^ km^2^/6) ∼ 305,000 species of arthropods in Amazonia. Here we have assumed that the small-scale data from the SLPA are applicable to the Amazon, but of course a better estimate would be obtained if our original data base were from regions within Amazonia, not Panama. Our calculation is only intended to be illustrative of an approach. For a review of other approaches to upscaling tropical arthropod species richness, see [19].

Three additional brief topics for discussion concern the relationship between distance decay and upscaling species richness, the validity of the predicted SAD at spatial scales larger than the censused plots. and the sensitivity of the results of upscaling to the choice of taxonomic units.

We mentioned in Results that our upscaling results within the Reserve are at least qualitatively consistent with observed distance decay of species similarity. The rate of distance decay of species similarity can vary considerably with habitat and taxa (see, for example, [20]), and this may eventually provide an opportunity to carry out further tests and applications of our MaxEnt-based predictions. But currently we have not been able to predict the rate of distance decay from MaxEnt, nor relate it to the shape of the SAR. This is an area of ongoing research.

Secondly, the form of the SAD at large spatial scales is the subject of legitimate debate [11,21], and in particular the relative merits of the log series versus the lognormal function. Our MaxEnt procedure predicts a log series SAD [10], as does the metacommunity prediction from neutral theory [7]. While our up-scaling predictions are relatively insensitive to small deviations from the predicted log series function, a model that predicts far less rarity than does the log series at large spatial scales could yield much lower estimates of species richness at large scale. At least for trees, the success of our methods both for the Western Ghats (60,000 km^2^) and for Panama (80,000 km^2^) provides some assurance that our theory is applicable at large scales.

Finally, in response to recent concern about sensitivity of the predicted SAR to the choice of taxonomic units [22, 23,24], we have shown that the predictions are relatively insensitive to the division of species into guilds but more sensitive to the division of species into taxonomic orders. The discrepancy of 31% in upscaled predictions at the scale of Panama is not insignificant, but we suggest that uncertainties at this level are likely also found in the empirical measurements of *S* and *N* that form the starting point of our analysis. For comparison, the standard error on our arthropod predictions at this scale is approximately 10% and the discrepancy between predicted total richness in a single area of 0.48 ha and measured richness in the twelve disjoint 0.04 ha plots is approximately 50%.

## Conclusion

A MaxEnt-based inference procedure predicts over 22,000 arthropod species in a 6000 ha tropical forest reserve using only census data obtained from twelve thoroughly censused 0.04 ha plots as the empirical input to up-scaling. Our results indicate that focal arthropod diversity in the reserve is more than twice that originally inferred by Basset et al. [1]. Previous evidence [4] from a single test of the up-scaling procedure in the Western Ghats and numerous smaller-scale tests of the predicted form of the SAR, which is the basis for the up-scaling method, augment confidence in our up-scaling procedure. Further support comes from the excellent agreement between empirical and inferred tree species diversity at the scale of Panama. Nevertheless, additional testing of the up-scaling procedure, in locations where the true answer is at least approximately known, will augment confidence in the approach. Finally, we have also suggested a procedure, admittedly speculative, by which our approach may be applicable to yet larger scale species richness estimations, even up to the scale of large biomes such as Amazonia, where it yields an estimate of 305,000 arthropod species.

## Supporting Information

S1 Script Python code to perform upscaling analysis. (PY)

S1 Table Detailed upscaling results separated by arthropod order, arthropod guild, arthropod plot, and tree plot. (XLSX)

S1 Text Summary of Maximum Entropy Theory of Ecology and additional methods. (DOCX)
