# Supplementary material for: Inferring Regional-Scale Species Diversity from Small-Plot Censuses
Source: PLoS One. 2015 Feb 23;10(2):e0117527. doi: 10.1371/journal.pone.0117527 (PMC4338294; doi:10.1371/journal.pone.0117527)
Supplement: S1 Text — (DOCX) [file pone.0117527.s003.docx]

**Supplementary Information**

**Summary of Maximum Entropy Theory of Ecology**

We have previously described a Maximum Entropy Theory of Ecology that predicts many important ecological patterns from a set of four “state variables” describing a community: the number of species in the community, *S*_0_, the total number of individuals across all species, *N*_0_, the total energetic requirements of all individuals, *E*_0_, and the area in which the community is found, *A*_0_. While this theory is described in detail in several previous publications [S1-S3], we summarize here the principle of maximum entropy on which the theory is based, the core equations of the theory, and the specific predictions of the theory with regard to species abundance and distribution.

In his work on information theory, Claude Shannon [S4] proposed a metric *H* that he called information entropy. Given a probability distribution *p*_i_, the information entropy of the distribution is given by:

$H=-\sum_{i=1}^{N} p_{i}\ln{(p}_{i})$ (Eq. S-1)

The index *i* is the independent variable that the probability function *p* depends upon, and so if *p* is the species abundance distribution, *i* refers to abundance. *H* is a measure of the degree of remaining uncertainty about the result of a draw from the distribution when the shape of the distribution is known. Thus if *p_i_* is sharply peaked *H* is relatively small while if *p*_i_  is flatter, *H* is larger.

Jaynes [S5] proposed that the best possible inference for an unknown probability distribution is the distribution that maximizes its information entropy, subject to any known constraints (such as a known mean) on the distribution. Jaynes showed that any distribution that does not maximize information entropy, subject to the prior knowledge that constitutes the constraints, must implicitly assume additional information that is not warranted by prior knowledge and thus represents bias.

Applying the principle of maximum entropy (MaxEnt) to obtain the “least biased” probability distribution, subject to known constraints, thus requires maximizing *H* subject to *k* constraints on the expected value of the distribution that can be written as

$\sum_{i=1}^{N} f_{k}\left( i \right)p_{i}=c_{k}$ (Eq. S-2)

where $f_{k}\left( i \right)$ is an arbitrary function of *i* whose known expectation is given by $c_{k}$. For example, if the mean of the distribution is known, this constraint can be written as $f_{k}\left( i \right)=i$ and $c_{k}=\mu$. A normalization constraint for the *p*_i_, which can be written as $f_{k}\left( i \right)=1$ and $c_{k}=1$, is additionally imposed.

Constrained maximization is carried out using the technique of Lagrange multipliers, which yields the general MaxEnt solution:

$p_{i}= \frac{e^{-\sum_{k=1}^{K} \lambda_{k}f_{k}(i)}}{Z}$ (Eq. S-3)

where *K* is the number of constraints and the partition function *Z* is given by

$Z = \sum_{i=1}^{N} e^{-\sum_{k=1}^{K} \lambda_{k}f_{k}(i)}$ (Eq. S-4)

The $\lambda_{k}$ are Lagrange multipliers that can be solved numerically using the solutions above and the constraint equations.

The Maximum Entropy Theory of Ecology [METE, S1-S3] is based on the application of this principle to two distributions. The first distribution, $R(n,\epsilon)$, gives the joint probability $R(n,,\epsilon)$d$\epsilon,$ that a randomly selected species has abundance *n* and that a randomly selected individual from that species has a metabolic energy requirement in the interval $(\epsilon,\epsilon+d\epsilon)$.

In a system described by the three non-spatial state variables *S*_0_, *N*_0_, and *E*_0_, the distribution $R\left( n,\epsilon\right)$ is subject to the three constraints: a normalization constraint, the constraint on the mean number of individuals per species, and the constraint on the mean energy per species.

Subject to these constraints, the general solution for $R\left( n,\epsilon\right)$ is given by

$R(n,\epsilon)= \frac{e^{-\lambda_{1}n-\lambda_{2}n\epsilon}}{Z}$ (Eq. S-5)

where $\lambda_{1}$ and $\lambda_{2}$ are Lagrange multipliers that are numerically determined from the constraint equations.

The species abundance distribution $\phi\left( n \right)$, giving the probability that a randomly selected species has *n* individuals, is found by integrating the $R\left( n,\epsilon\right)$ distribution over the $\epsilon$ variable. In doing so, the species abundance distribution is found to be approximately equivalent to an upper truncated Fisher logseries distribution with support extending to $N_{0}$ (as no single species can have more than $N_{0}$ individuals). This distribution can be written in simplified as

$\phi\left( n \right)=\frac{ce^{-\left( \lambda_{1}+\lambda_{2} \right)n}}{n}$ (Eq. S-6)

where *c* is a normalization constant.

The second key distribution of the Maximum Entropy Theory of Ecology is the species-level spatial abundance distribution, $\Pi(n)$ which gives the probability that an individual species with total abundance *n*_0_ in *A*_0_ has abundance *n* in a randomly selected cell of area *A* within *A*_0_. Given the necessary normalization constraint and the constraint that the mean of this distribution must equal *n*_0_ *A* / A_0_, $\Pi(n)$ is predicted to be an upper truncated geometric distribution with support extending to *n*_0_.

$\Pi(n) = \frac{e^{-\lambda_{\Pi}n}}{Z}$ (Eq. S-7 )

where $\lambda_{\Pi}$ is a Lagrange multiplier that can be calculated using the constraint equation.

The species abundance distribution and the species-level spatial abundance distribution can be combined to yield an expression for the species area relationship, in which the expected number of species in an area *A* is the product of *S*_0_ and the probability that a randomly selected species is present in *A*. This second term is given by the sum over the product of the probability that a species has a total abundance *n*_0_ in *A*_0_ and the probability that a species with total abundance *n*_0_ is present in a cell of area *A*.

$S=S_{0}\sum_{n_{0}=1}^{N_{0}} [1-\Pi\left( 0|n_{0} \right)]\phi(n_{0})$ (Eq. S-8)

This expression for the species area relationship can be used to upscale species richness based on small scale census data. Define now *S*_0_, *N*_0_, and *A*_0_ as the census scale, where all three of these state variables are known, and *S*_1_, *N*_1_, and *A*_1_ as the state variables at a larger scale such that *A*_1_ = 2*A*_0_. Because the estimated total number of individuals scales linearly with area (because we are using a completely nested design), the scaling procedure aims to estimate the unknown state variable *S*_1_.

For the special case of doubling areas, this problem reduces to solving two equations, the equation for *S*_1_ given above and the constraint equation that solves for the $\lambda_{i}$, which contain only those two unknowns (for the special case of doubling area, the additional unknown parameter $\lambda_{\Pi}$ cancels out of the expression for $\Pi(0)$). Once the value of *S*_1_ is known, this procedure can be iterated to successively higher doublings of area. The iteration yields the predicted curves in Figures 1 and 3 of main text. Detailed derivations of all results above are provided in ref. S3.

**Additional Methods**

Our analysis of species abundance distributions was completed using the open-source package *macroeco* v0.2 (<http://github.com/jkitzes/macroeco>). Upscaling analysis was conducted using the Python script included in Supporting Information (Script S2).

Comparisons of observed and predicted rarity for plot-order and plot-guild combinations were carried out by calculating the *R*^2^ around a one-to-one line (S6), which gives the proportion of variation in the observed data that is explained by the theoretical predictions. This *R*^2^ calculation used the equation

$R^{2}=1-\frac{{\sum_{i=1}^{N} (n_{i,obs}-n_{i,pred})}^{2}}{{\sum_{i=1}^{N} (n_{i,obs}-\bar{n}_{i,obs})}^{2}}$ (Eq. S-9)

where *n*_i_ is the *i*-th plot-order or plot-guild combination and *N* is the total number of plot-order or plot-guild combinations.

**References**

S1. Harte J, Zillio T, Conlisk E, Smith A (2008) Maximum entropy and the state-variable approach to macroecology. Ecology 89: 2700–2711.

S2. Harte J, Smith AB, Storch D (2009) Biodiversity scales from plots to biomes with a universal species-area curve. Ecol Lett 12: 789–797.

S3. Harte J (2011) Maximum Entropy and Ecology: A Theory of Abundance, Distribution, and Energetics. Oxford: Oxford University Press. 264 p.

S4. Shannon CE (1948) A Mathematical Theory of Communication. Bell Syst Tech J 27: 379–423.

S5. Jaynes ET (1982) On the rationale of maximum entropy methods. Proc Instit Elec Electron Eng 70: 939-952.

S6. White EP, Thibault KM, Xiao X (2012) Characterizing species abundance distributions across taxa and ecosystems using a simple maximum entropy model. Ecology 93: 1772–1778.
